# Aptamer-nanobody based ELASA for detection of *Vibrio cholerae* O1

**DOI:** 10.18502/ijm.v12i4.3928

**Published:** 2020-08

**Authors:** Alireza Ebrahimi Mojarad, Seyed Latif Mousavi Gargaria

**Affiliations:** Department of Biology, Shahed University, Tehran, Iran

**Keywords:** Aptamer, *Vibrio cholerae*, Ligands, Flow cytometry

## Abstract

**Background and Objectives:**

In recent years, the prevalence of diseases caused by *Vibrio* spp. is increasing in the world, and among them species, *Vibrio cholerae* is the most important *Vibrio* associated with pandemic and epidemic cholera outbreaks. Therefore, the development of a reliable method for early and accurate detection of *V. cholerae* for management of diseases is a real need. Aptamers with the ability to detect targets with high specificity and accuracy can be one of the candidates used for the whole cell and thereby *V. cholerae* detection.

**Materials and Methods:**

In this research high-affinity DNA aptamers against with two major serotypes of Inaba (ATCC 39315) and Ogawa (clinical sample) were selected from DNA aptamer library through 12 rounds of Systematic Evolution of Ligands by Exponential (SELEX) enrichment procedure using live cells as a target which monitored with flow cytometry.

**Results:**

The binding efficiency and dissociation constant of the isolated aptamers V.ch47 and V.ch27 were 56.4%, 53.3% and 15.404 ± 4.776 pM, 20.186 ± 3.655 pM, respectively. A sandwich Enzyme-linked aptamer sorbent assay (ELASA) was developed with the biotinylated V.ch47 aptamer and our previously developed nanobody anti-Lipopolysaccharides (LPS). We optimized this system with *V. cholerae* O1 and analyzed their cross reactivity with close physiological bacteria. The threshold of detection was obtained 10^4^ CFU/ml in the sandwich ELASA process.

**Conclusion:**

Our results showed that the sandwich ELASA is sensitive enough for the rapid detection of *V. cholerae* from other bacteria.

## INTRODUCTION

*Vibrio cholerae* is a Gram-negative, non-spore forming curved rod bacterium with polar flagella found mostly in marine environments. It is classified based on its major surface antigens into nearly 206 serogroups, of which O1 and O139 cause epidemic cholera. Cholera is an infectious disease characterized by intense vomiting and watery diarrhea which if left untreated, could lead to harsh dehydration and death (1). The ability of *V. cholerae* to survive in water supplies, aquatic ecosystems and inappropriate sewage disposal and short incubation period of the disease play a significant role in cholera epidemics. While only 2–5 % of *V. cholerae* infections will result in severe diarrhea; roughly 75% of them are asymptomatic. Cholera symptoms might start before 2 h or after 5 days infection and in severe disease could make to loss of up to 2 liters of water per hour, which may result in death within 24 h. Cholera outbreaks happen at regular intervals and it’s still endemic in some countries. Furthermore, there have been seven pandemics since 1817 and it’s still known as one of the significant health threats, especially in developing countries. Whereas *V. cholerae* O139 has been causing some limited number of outbreaks in the world recently, all the cholera pandemics initiated by O1 serotype (2, 3). The gold standard for detection of *V. cholerae* from water is a culture method, but this requires laboratory infrastructure, besides it is time-consuming, especially in management a large number of water samples. There are some rapid detection methods, including nucleic acid-based methods such as PCR, immunomagnetic beads and DNA probe hybridization. The sensitivity of immunomagnetic bead is low and DNA probe hybridization and the PCR techniques require designing specific probes or primers both based on the detection of nucleic acids rather than live bacterial cells increasing the possibility of false positives results (4). Gubala et al. (2006) and Blackstone et al. (2007) developed a real-time PCR technique for detecting *V. cholerae* with high sensitivity. This technique is also based on nucleic acid detection and still requires isolation and culture of the sample after PCR confirmation (5, 6). In 2006, surface plasmon resonance immunosensor was developed to target *V. cholerae* O1 using a monoclonal antibody. Their detection range was between 105 and 109 cells/ml (7). Chakraborty et al. introduced a rapid dipstick test based on the detection of LPS antigen directly from fecal samples. The test was shown to be sensitive >90% and roughly specific −70%; with limitations like not being able to detect Viable but nonculturable (VBNC) vibrios since the test relies on the ability of the bacteria to grow in the Alkaline peptone water (APW) culture medium or does not differentiate non-toxigenic from toxigenic strains; as the dipstick test detects the LPS of *V. cholerae* (8).

Aptamers are short (35–100 nucleotides) RNA or single-stranded DNA molecule, which can bind to specific targets due to their 3D conformations with high specificity. Aptamers are separated by *in vitro* screening and systematic evolution of ligands by exponential enrichment technology (SELEX). This is a high-throughput screening method that involves the selection of aptamers by repeated rounds of amplification and partitioning from a pool of random synthetic oligonucleic acid (9). Small size, cost-effectiveness, chemical stability and convenient and flexible structures of aptamers have led them to be used in highly selective and sensitive biosensors for the identification and detection of pathogens (10). Recently aptamers against several pathogens like *Listeria monocytogenes, Streptococcus mutans, Haemophilus influenzae* type b, *Staphylococcus aureus* and a mixture of GAS M-types by whole cell-SELEX technique have been characterized (11, 12). The whole cell-SELEX is preferred for the detection of pathogens because in the live bacterial cells, the aptamers are selected for the surface molecules in their natural conformations condition. In another word, the conformation of the target remains the same from selection to detection (13).

In this research, we isolated a specific DNA aptamer with a high binding affinity toward *V. cholerae* through whole cell-SELEX. We have previously developed an Anti-Lipopolysaccharides nanobody (LPS) of *V. cholerae* O1 (14). Using this nanobody along with our selected aptamer, we developed a new detection system (an aptamer and nanobody) for recognition of *V. cholerae* O1 in an ELASA based system.

## MATERIALS AND METHODS

### Bacterial strains, culture conditions and preparation of cells.

The *V. cholerae* O1 [Inaba (ATCC 39315) and Ogawa] used as targets were obtained from Shahed University. The other bacterial strains listed in Table 1 were used in cross-reactivity tests and counter-SELEX to raise the specificity of selection. All strains were verified with biochemical tests as well as amplification of 16S rRNAs (data not shown) and cultured under aerobic conditions in Luria Brentani (LB) broth at 37°C. For each round of SELEX, 10^8^ CFU/ml of *V. cholerae* O1 (200 μl ogawa and 250 μl inaba (OD600 nm= 0.4)) in logarithmic phase were taken, washed with wash buffer (1× PBS plus 0.02% tween 20) at 7000 ×g for 10 min; then suspended in 200 μl of binding buffer (wash buffer containing 1% bovine serum albumin (BSA) was used.

**Table 1.** Bacterial strains

**Bacterial cells**	**Collection center**
*Vibrio cholerae* O1, Inaba, ATCC 39315	
*Vibrio cholerae* O1, Ogawa^*^	
*Salmonella* Typhimurium^*^	Obtained from
*Salmonella* Enteritidis^*^	Microbial
*Shigella flexneri*, NTCC 12678	Collection
Shigella dysenteriae^*^	center of the
*Escherichia coli*, ATCC 25922	Shahed University
*Yersinia enterocolitica*, ATCC 23715	
*Enterobacter* (sp)^*^	
*Klebsiella pneumoniae*, ATCC:13883	
*Proteus vulgaris*, ATCC 6380	

* Clinical isolate

### Amplification of the aptamer library and preparation of ssDNA aptamers.

78-mer combinatorial dsDNA library procured from Metabion (Steinkirchen, Germany). Primers were synthesized at TAG Copenhagen A/S (Frederiksberg, Denmark). The sequences of DNA library, unlabelled and labelled primers are listed in Table 2. Aptamer library with an initial concentration of 10 μM was amplified in a 50 μl PCR reaction (Bio-Rad T100 Thermal Cycler, USA) by a three-step thermal protocol starting with an initial denaturation at 95°C for 5 min followed by 30 cycles of 95°C for 30 s, 62°C for 30 s, 72°C for 30 s, and a final extension at 72°C for 5 min. DNA library pools were melted to ssDNA by heating at 95°C for 10 min, then flash cooled in ice for 15 min. Converting dsDNA to ssDNA proved by size separation on 10% denaturing urea PAGE. The concentration of oligonucleotides was measured with Pico drop 200 (Pico Hinxton, United Kingdom).

**Table 2.** Oligonucleotides used in SELEX and ELASA

**Name**	**Oligonucleotides**
Aptamer library	5′-GCCTGTTGTGAGCCTCCTAAC(N38)CATGCTTATTCTTGTCTCC-3′
Forward -primer	5′-GCCTGTTGTGAGCCTCCTAAC-3′
Reverse -primer	5′-GGAGACAAGAATAAGCATG-3′
FITC-forward primer	5′-FITC-GCCTGTTGTGAGCCTCCTAAC-3′
Biotin-forward primer	5′-Biotin-GCCTGTTGTGAGCCTCCTAAC-3′

### Aptamer selection process (SELEX).

To start the first round of SELEX, 2 nmol ssDNA library was mixed with 200 μl of 1× binding buffer and then added with 10^8^ CFU/ml of target cells. The reaction mixture was incubated for 1 h at 4°C with gentle shaking. The cells were recovered by centrifugation at 7000 ×g at 4°C for 10 min. Cells were washed to eliminate unbounded/weakly bounded ssDNA aptamers and collected by centrifugation at 7000 ×g for 10 min. Cells were suspended by adding 70 μl of 1× PCR reaction buffer and heated at 70°C for 12 min. After centrifugation the supernatant containing bounded aptamers was used as a template and amplified with PCR to be used for the next rounds of SELEX. Some modifications were applied in PCR protocol for amplification of cell-bound aptamers after each SELEX. To strengthen the binding condition, the washing frequency was increased from SELEX 2 onward with ascending concentration of tween 20 (0.02% to 0.07%). Simultaneously the incubation time was gradually decreased from 60 min in the first to 15 min in the last round of SELEX. A total of twelve rounds of SELEX and three rounds of counter-SELEX (3^rd^, 7^th^ and 8^th^ rounds) were performed to select high-binding aptamers toward *V. cholerae* O1. In counter-SELEX we also used 10^8^ CFU/ml of bacterial cocktail. The SELEX procedure was similar to that of positive SELEXs. The supernatant containing unbounded aptamers was used as a template to amplify and use in the next round of SELEX.

Bacteria used for the counter-SELEX were: *E. coli* O157:H7, *Shigella dysenteriae, Salmonella enteritidis, Salmonella* Typhimurium, *Yersinia* and *Shigella flexneri.*

### Flow-cytometric analysis of aptamer binding affinity.

The binding efficiency of ssDNA aptamer pool toward target cells, SELEX steps were monitored with flow cytometry using FITC-labeled primers. The outcome of 2^nd^, 5^th^, 10^th^, 11^th^, 12^th^ SELEXs and initial ssDNA library were amplified and fluorescence intensity was assessed using Attune NxT (Thermo Fisher, USA). In brief, incubation of 50 pM of FITC labeled aptamer with 10^8^ CFU/ml of target cells in binding buffer, followed by incubating at 4°C for 15 min. Cells were washed with washing buffer, harvested with centrifugation and resuspended in 0.5 ml binding buffer. The binding affinity was measured by the flow cytometer.

### Cloning procedure.

Aptamers obtained from the last round of SELEX were amplified by PCR and were cloned into a pTG19-T vector (Vivantis, Selangor, Malaysia). The ligation reaction was transformed into *E. coli* DH5α competent cells (Thermo Fisher Scientific, Waltham, MA USA) and plated on LB agar containing 50 μg.ml^−1^ ampicillin. Colonies were analyzed with colony PCR. Each positive clone was amplified with FITC-labeled primers and the fluorescence intensity was estimated by flow cytometer. Then aptamer candidates with the highest binding affinities were selected for further analysis.

### Affinity measurement of the bounded aptamer.

To study the cross-reactivity of selected aptamers, individual samples of *Enterobacter, Klebsiella* and *Proteus vulgaris* were tested for cross-reactivity with the same procedure described above. In brief, 10^8^ CFU/ml of the bacteria were mixed with 50 pM of the fluorescently labeled high binding aptamers. The florescent intensity of the samples were measured with flow cytometry. Two aptamers with the highest affinity to *V. cholerae* and minimum affinity to other bacteria were chosen and sent for sequencing (Tehran University, Iran). The sequences were analyzed with CLC Sequence Viewer 8.0. For determination of the equilibrium dissociation constant (Kd), different concentrations from each aptamer (0, 50, 100, 150, 200 and 250 pM) were mixed with 10^8^ CFU.ml^−1^ of *V. cholerae* O1 in binding buffer and incubated at 4°C for 45 min with gentle rotation. The mixtures were washed before subjecting to flow cytometry (15, 16). The saturation curve was plotted and Kd of aptamers were calculated with non-linear regression analysis by Sigma Plot 14.0 and the equilibrium dissociation constant (Kd) was calculated (13).

### Sequencing and structural analysis of selected aptamers.

Two aptamers with the highest affinity toward *V. cholerae* and minimum affinity to other bacteria were sequenced (Tehran University, Iran). The secondar y structure of the selected aptamer sequences was predicted using the online software Oligo Analyzer3.1 (https://www.idtdna.com/calc/analyzer). The Prediction of structural folding was carried out under the circumstances set up of 144 mM Na^+^ at 21°C.

### ELASA based detection of *V. cholerae.*

ELASA assay was carried out for detection of *V. cholerae* O1 (*Inaba* and *Ogawa*) using anti-LPS single-domain antibody fragments (VHHs) for *V. cholerae* O1 recognizing Inaba and Ogawa serotypes as capture and biotinylated aptamer V.ch47 as a detector. *Salmonella typhimurium, Salmonella enteritidis, Shigella flexneri, Shigella dysenteriae, E. coli* O157:H7, *Yersinia, Enterobacter, Klebsiella* and *Proteus vulgaris* were used for assessment of any possible cross-reactivity. All the reactions were repeated 3 times.

The expression and purification of VHH were done as described previously (14). 100 μl of VHH (20 μg.ml^−1^) in carbonate–bicarbonate buffer (pH 9.5) was coated on the microplate and incubated over-night at 4°C. Wells were washed three times with PBS-T (PBS + 0.05% Tween 20) and unbound locations were blocked with 100 μl skim milk at 37°C for 1 h and washed three times with PBS-T. The bacterial cocktail having 10^8^ CFU.ml^−1^ was added to the wells and incubated at 37°C for 2 h. After washing with PBS, 100 μl of biotinylated aptamer V.ch47 was added to the wells and incubated at RT for 2 h. Wells were washed and 100 μl of 1:1000 dilution of horse-radish peroxidase (HRP) conjugated with streptavidin (Biolegend, San Diego, USA) was added to the wells and incubated at 37°C for 90 min, then washed with PBS. Finally, 100 μl of tetramethylbenzidine (DNAbiotech Co., Iran) as a substrate was added. The plate was incubated at room temperature (RT) for 10 min in a dark condition. The reaction was stopped with 100 μl H_2_SO_4_ 3N and the OD450 was measured using Infinite F50 microplate reader (TECAN, Mannedorf, Switzerland). Cross-reacting Bacteria was also used for negative control. The same experiment was carried out with different enumeration of bacteria (10^4^–10^8^ CFU/ml) and different concentrations of aptamer (25, 50 and 100 pM), to obtain the limit of detection (LOD).

## RESULTS

### SELEX optimization and flow cytometry.

**Fig. 1.**
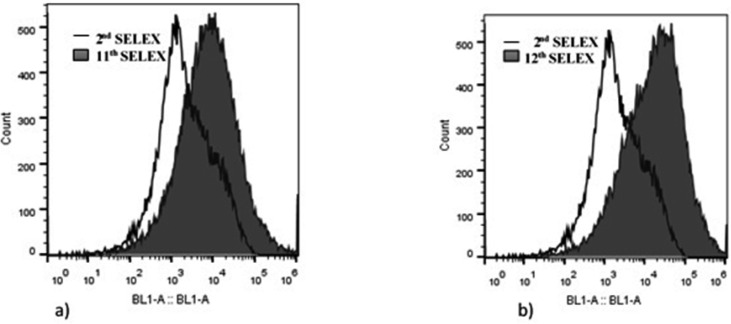
Results obtained from flow cytometry shows increased binding affinity of 11^th^ (a) and 12^th^ (b) rounds of SELEX compared to that of 2^nd^ round. The binding efficiency increased from 14.7% in the 2^nd^ round to 50.3% and 50.9% in the 11^th^ and 12^th^ rounds respectively.

Twelve rounds of SELEX including nine positive-SELEX and three counter-SELEX were carried out to reach the saturation state. The fluorescent intensity of the ssDNA aptamer pool bound to the target was increased from 14.7% in the second round to 50.3% and 50.9% in the 11^th^ and 12^th^ rounds of SELEX, respectively (Fig. 1). The binding efficiency during the last two SELEX rounds (11^th^ and 12^th^) didn’t show any impressive growth; therefore, the SELEX process was terminated at round 12^th^.

Our data revealed descending binding affinity toward counter bacteria and the results of SELEX 12^th^ showed approximately 4-fold improvement in binding efficiency compared to the 2^nd^ round. The ssDNA aptamer pool of the latest round was cloned and transferred into the *E. coli* DH5α cells. Thoroughly 97 clones were achieved, of which 67 aptamer transformants were confirmed as positive clones with colony PCR. The binding affinity of 31 positive clones to target bacteria was analyzed (Table 3). In the next step flow cytometer determined the binding efficiency to cross bacteria (Table 4).

**Table 3.** Fluorescent intensity of 31 selected aptamers to *V. cholerae* O1

**Aptamer name**	***V. cholerae* O1**	**Aptamer name**	***V. cholerae* O1**
	
	**Fluorescence intensity (%)**		**Fluorescence intensity (%)**
v.ch1	42.2	v.ch33	42.9
v.ch2	52.7	v.ch35	29.1
v.ch3	52.6	v.ch36	44.1
v.ch6	38.1	v.ch37	42.8
v.ch8	42	v.ch38	46.1
v.ch10	49.1	v.ch42	45.5
v.ch11	44.9	v.ch44	51.2
v.ch12	16	v.ch47	56.4
v.ch15	50.8	v.ch48	41.3
v.ch17	39.9	v.ch50	52.5
v.ch18	49.3	v.ch53	47.4
v.ch20	53	v.ch55	49.7
v.ch24	28	v.ch56	51.2
v.ch25	24	v.ch61	47.1
v.ch27	53.3	v.ch67	48.5
v.ch29	25.9	-	-

**Table 4.** Affinity analysis with cross bacteria

**Aptamer name**	***V. cholerae* O1**	***Enterobacter***	***Klebsiella***	***P. vulgaris***

	**Fluorescence intensity (%)**			
V.ch27	53.3	3.32	5.57	4.56
V.ch47	56.4	5.14	17.90	9.25
V.ch50	52.5	3.03	10.90	4.03

**Fig. 2.**
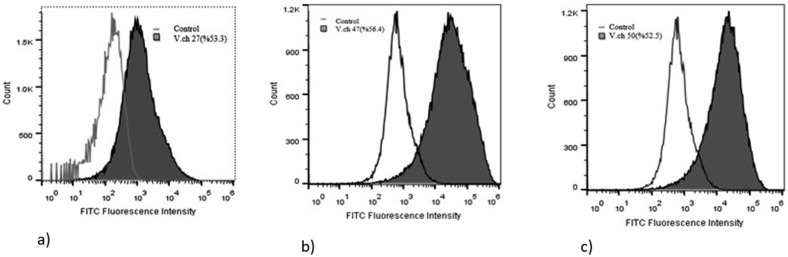
Binding affinity of the three high binders to target bacteria. The binding efficiency of aptamers V.ch27 (a), V.ch47 (b) and V.ch50 (c) were 53.3%, 56.4% and 52.5% respectively.

Finally, three aptamers with the highest affinity to *V. cholerae* O1 and the lowest affinity to other bacteria used for cross-reactivity were selected for further analysis. The affinity of 3 aptamers for the target were estimated as 56.4%, 53.3% and 52.5% and here onward named as V.ch47, V.ch27 and V.ch50, respectively (Fig. 2).

### Dissociation constants.

**Fig. 3.**
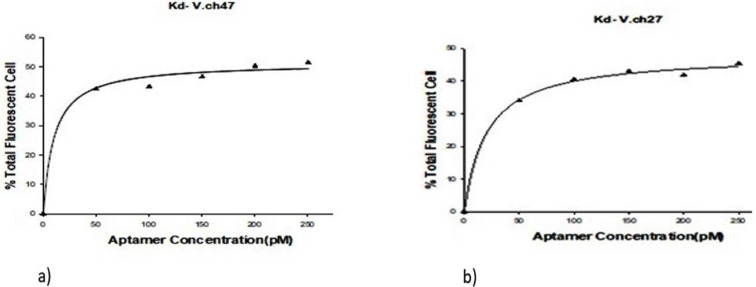
Dissociation constant curve of V.ch47 and V.ch27 aptamers analyzed with y=Bmax × x/ [kd + x] equation. The yielded Kd values were 15.404 ± 4.776 pM for V.ch47 and 20.186 ± 3.655 pM For V.ch27.

The percent binding efficiency between the aptamer and target cells was estimated by considering a constant amount of 10^8^ CFU/ml^−1^ of *V. cholerae* O1 cells against varied aptamer concentrations from 0 to 250 pM. The dissociation constant values (Kd) of V.ch47 and V.ch27 were calculated as 15.404 ± 4.776 and 20.186 ± 3.655 pM, respectively. The related binding saturation curve is displayed in Fig. 3.

### Sequence analysis of selected aptamers.

**Fig. 4.**
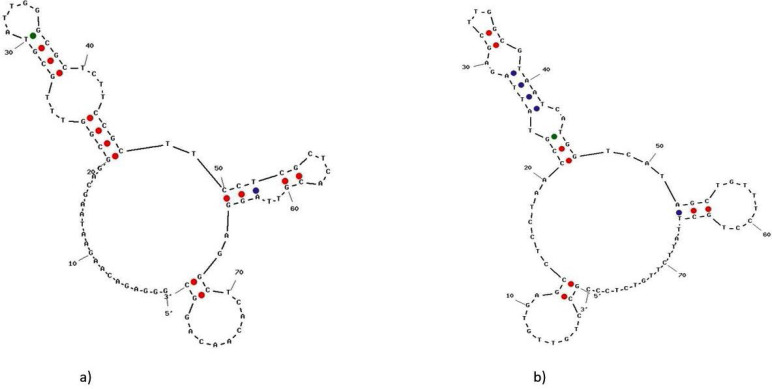
Predicted secondary structure of selected aptamers. The range of free energies were −13.21 to −16.31 (kcal/mole) for (a) V.ch27 and −6.41 to −8.62 (kcal/mole) for (b) V.ch47.

The sequence of the variable regions of the selected aptamers is given in Table 5. The Gibb’s free energy of each sequence was calculated by the UNAFold online program. The secondary structures were predicted at 21°C with a minimum free energy of 16.39 kcal.mol^−1^ for aptamer V.ch27 and −8.62 kcal.mol^−1^ for aptamer V.ch47 are displayed in Fig. 4.

**Table 5.** The ssDNA aptamer sequences with highest binding affinity

**Aptamer No.**	**Sequence (without primers)**	**dG value (kcal/mol)**
V.ch27	GGCGGTTTGCGTATTGGGCGCTCTTCCGCTTCCTCGCTCAC	−16.39
V.ch47	CGTATTAGAGCTTGGCGTAATCATGGTCATAGCTGTTTC	−8.62

### Detection of *V. cholerae* O1 using V.ch47 aptamer in ELASA.

**Fig. 5.**
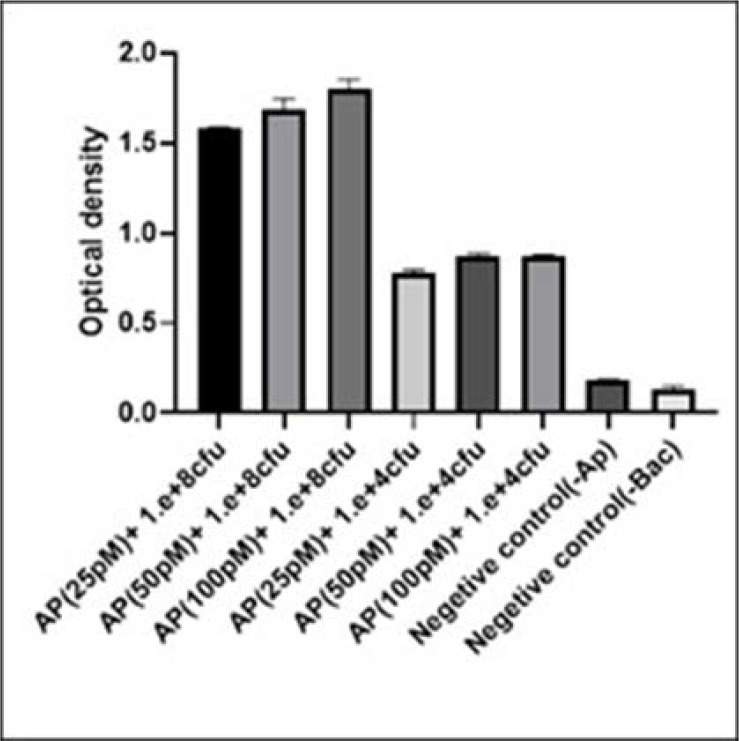
Determination of the cut-off value for *V. cholerae* O1 in the ELASA test. Two different concentration of bacteria (10^4^ and 10^8^ CFU.ml^−1^) with three different concentration (25, 50, 100 pM) of aptamer V.ch47 were tested in ELASA. The experiment was conducted with two negative controls, one without aptamer and one without bacteria. The bacterial concentration (CFU.ml^−1^) is shown on the horizontal axis. Error bars display the standard deviation of the OD_450_ mean of three experiments.

ELASA planned as a pentamerous complex (VHH-bacteria-biotinylated V.ch47-streptavidin HRP conjugate and TMB) with three wells repetition. The results of ELASA is displayed in Fig. 5. The value of reaction containing 10^4^ CFU. ml^−1^ *V. cholerae* O1 and 25 pM ssDNA aptamer became nearly 0.77, was selected as an optimum readout for further analysis. Four different bacteria are selected to investigate the cross-reactions and the results are exhibited in Table 6. There was a significant difference between the optical density gained from *V. cholerae* O1 and other bacteria used as a cross. The OD_450_ obtained for *V. cholerae* O1 was 0.77, while the highest OD_450_ related to other bacteria was 0.3 belonging to *S. flexneri* (Table 6). The optical density of the reactions in the absence of aptamer/bacteria was always less than 0.2.

**Table 6.** ELASA results of *V. cholerae* O1 and bacteria used for cross-reactivity. Results are displayed as an OD_450_ mean ± standard deviation (n=3)

**Bacteria (10**^**4**^ **CFU/ml)**	**OD**_**450**_
*Vibrio cholerae* O1	0.7629 ± 0.001
*Shigella flexneri*	0.2924 ± 0.029
*Enterobacter*	0.1788 ± 0.04
*Proteus volgaris*	0.2297 ± 0.015
Negative Control (-bacteria)	0.0992 ± 0.001
Negative Control (-Aptamer)	0.112 ± 0.005

## DISCUSSION

*V. cholerae* which causes an epidemic infectious disease of cholera, generally spread in marine environment. This bacterium is mostly present in water supply environments of cholera patient’s existence (1). WHO has estimated, having 1.3 million to 4.0 million cases of cholera, and 21000 to 143000 deaths in the world annually. Moreover, outbreaks continued to impress numerical countries. Cholera often affects developing countries with no proper contact with suitable water and sanitation resources and thereby remains a major public health problem yet. Although the O1 and O139 serogroups are related to epidemic disease, *V. cholerae* O1 was the main cause of all recent outbreaks. Therefore rapid detection of *V. cholerae* O1 in polluted waters or clinical samples like stool; in early stages is critical, mainly for the prevention of the outbreaks (17).

In recent years, aptamers and recombinant antibodies have been used in different diagnostic systems such as ELASA, blotting assay, flow cytometry, immunocytochemistry, immunofluorescence microscopy and microarray (18, 19). In the present work, we have developed an ELASA system for detection of *V. cholerae* O1, where nanobody and aptamer, both developed specifically against *V. cholerae* O1, were used as capture and detector respectively.

Aptamers are widely used for the detection of bacteria with SELEX procedure where they show high specificity and binding affinity toward their targets (13, 16). In this research, a whole-cell SELEX method was used to isolate ssDNA aptamers for *V. cholerae* O1. Theoretically, the whole-cell SELEX allows candidate aptamers to recognize multiple cell surface targets for binding in their native conformation. The cell-SELEX approach was analyzed by flow cytometry for both binding affinity and selection. In the traditional protein SELEX, on the other hand, one cannot firmly determine the aptamer binding site in a physiological condition, and this may create problem with post-selection where the selected aptamer/aptamers may not bind to their targets on the cell surface (20).

V.ch47 and V.ch27 aptamers showed affinity of 15 ± 4 and 20 ± 1 pM toward *V. cholerae* O1 respectively. The data are in agreement with our and other previous reports, which indicate the efficiency of our selection process (11, 13, 21). The best Kd obtained was 15.404 ± 4.776 pM, characterized after 12 rounds of SELEX. Compared to our previous reports, for *Haemophilus influenzae* type b, *Streptococcus pyogenes* type M3 and *Acinetobacter baumannii*; the Kd for *V. cholerae* O1 was somewhat higher (18, 22). The reason could be due to having two serotypes as a target instead of having a single one in the SELEX procedure. But in comparison to other mention works, our selected aptamers showed better affinity (11, 13, 23). The higher affinity obtained here can be attributed to suitable selection stringency and using bacterial strains with similar physiological properties to those used in counter-SELEX, which considerably eliminates a non-specific, poor binding and non-target cells. Furthermore, amplification of the whole DNA pool after each SELEX helps us to reduce the possibility of losing any sequences (possibly high binder). Finally, the V.ch47 aptamer showed the highest specificity toward *V. cholerae* O1 (Inaba and Ogawa) with no affinity toward other related bacteria used in the cross and counter-SELEX exhibited in Table 1. RNA aptamers have also been selected against whole cell bacteria such as *E. coli* O157:H7(24); however RNA aptamers are harder to produce, less stable compared to DNA aptamers, and become cost-effective if modified in order to make them RNase-resistant. Yu et al. accomplished a total of 25 rounds of selection against live *E. coli* O157:H7. Their selected DNA aptamer exhibited a Kd of 10.30 nM (25)., whereas we obtained better a kd after 12 rounds of SELEX, which can be due to proper selection performance and the binding buffer used in procedures.

The affinity of the VHH against *V. cholerae* O1 LPS (lipopolysaccharide) used in ELASA was 5 × 10^−8^ M, described previously (14). Generally in the sandwich ELASA, either aptamer or antibody is fixed as a capture (26). The nanobody was immobilized on the surface of the wells because aptamer has smaller size compared to antibodies and fixing aptamer as a capture substance may modify the structural conformation of the aptamer which subsequently affects its binding to the target on the cell surface. Moreover, aptamer labeling is easier and does not affect their conformation, which makes them more suitable detectors compared to the antibodies (27). Our method was evaluated in numerical steps to verify the specificity and efficiency of the technique with different concentrations of target cells and aptamer. We could identify 10^4^ CFU.ml^−1^ (OD_450_ = 0.762) with 25 pM of biotinylated aptamer and only 20 μg.ml^−1^ of VHH without any cross-reactivity. Wang et al. generated an aptamer against prion protein and developed a sandwich ELISA with 0.5 ng.ml^−1^ aptamer as a capture and 2 ng.ml^−1^ prion, using antibodies in a common sandwich ELISA technique (28). In 2009, Chen et al. developed an ELASA system for the detection of Hepatitis C virus. They used aptamer as a detector and a 250 nM polyclonal antibody as a capturing agent (29). We previously designed an ELASA system for the detection of *A. baumannii* (ATCC 19606) by a biotinylated aptamer and a nanobody against biofilm-associated protein (Bap) in 2016. The detection threshold in our sandwich ELASA for *A. baumannii* was 103 CFU/ml (18). Better cut off value in the ELASA system for *A. baumannii* could be due to the development of higher affinity aptamers (7.547 ± 1.353 pM) and nanobody (3.8 × 10^−8^ M/L). Whereas in the present work, the Kd for aptamer and VHH were 15.404 ± 4.776 pM and 5 × 10^−8^ M, respectively (30). For detection of OmpW and CtxB antigens of *V. cholerae*, Tuteja et al. developed a dip-stick sandwich ELISA where the analytical sensitivity of the designed assay was 60 pg/ml for CtxB and 10^4^ CFU•ml^−1^ for OmpW (31). In another research, based on anti-lipopolysaccharide antibodies, Nato et al. designed an immunochromatographic method which able to detect 10^7^ CFU/ml^−1^ of O1 strain and 106 CFU/ml^−1^ of O139 (32). Also, Obihiro university designed a immunochromatographic test strip by Yamasaki et al. the reported detection limit was 10 ng/ml of cholera toxin (33). In the current research, Bayat et al. used IgY based on ELISA for the detection of toxigenic *V. cholerae* for the first time. They reported the limit of detection of OmpW is 10^3^ CFU/ml and that of CtxB is nearby 33 pg/ml (34) University of East Anglia developed a colorimetric bioassay method for Cholera toxin (CTB) with a limit detection of 54 nM (35). Lee et al. also used the whole-cell SELEX strategy to generate aptamers with high affinity against live *Listeria monocytogenes*. Their aptamer-based sandwich assay (ABSA) exhibited a linear response over an extensive concentration range of *L. monocytogenes* from 20 to 2 × 106 CFU/ml (18).

## CONCLUSION

In conclusion, we have introduced a specific and easy with high-affinity detection ELASA system based on a combination of V.ch47 aptamer and anti LPS nanobody; for detection of *V. cholerae* O1 without needing for robotic equipment or costly preparations. As we have mentioned previously, our ELISA system is flexible enough to be used in the detection of a wide range of pathogens.
